# First *in vivo* evaluation of the radiolabelled Hsp90 inhibitor [^11^C]Onalespib for cancer and brain PET imaging

**DOI:** 10.21203/rs.3.rs-10764394/v1

**Published:** 2026-08-28

**Authors:** Valeria Narykina, Romy Cools, Niels Van Winnendael, Koen Vermeulen, Guy Bormans, Ludovic Le Saux

**Affiliations:** 1 Laboratory for Radiopharmaceutical Research, Department of Pharmaceutical and Pharmacological Sciences, KU Leuven, 3000 Leuven, Belgium; 2 Switch Laboratory, VIB Center for Brain and Disease Research, Herestraat 49, 3000, Leuven, Belgium; 3 Switch Laboratory, Department of Cellular and Molecular Medicine, KU Leuven, Herestraat 49, 3000, Leuven, Belgium; 4 Nuclear Medical Applications, Belgian Nuclear Research Centre (SCK CEN), Mol, Belgium

**Keywords:** Cancer, diagnostic, glioblastoma, heat shock proteins, [^11^C]Onalespib, PET radiotracer

## Abstract

**Background:**

Heat shock protein 90 (Hsp90) is a key molecular chaperone involved in maintaining proteostasis, and its function and aberrant expression are linked to tumour progression and to neurodegeneration. Its overexpression in cancers and the clinical development of Hsp90 inhibitors, including the recently approved pimitespib (TAS-116, trade name Jeselhy^®^), highlights the need for non‑invasive imaging tools to assess Hsp90 expression. Current PET/SPECT tracers show limited performance. Onalespib, a clinically evaluated inhibitor with favourable chemical flexibility and identified as a promising radiosensitizer in glioblastoma, suggesting blood-brain barrier penetration, offers an attractive alternative. In this work, we report the first preclinical evaluation of [^11^C]Onalespib to assess its suitability as an Hsp90‑targeted PET radiotracer for tumour and brain imaging.

**Results:**

[^11^C]Onalespib was produced in high purity, with an appropriate radiochemical profile and a molar activity suitable for biological studies. *In vitro* autoradiography demonstrated strong Hsp90‑specific binding in U‑87 MG, MDA‑MB‑231, PC-3 tumour tissues and mouse brain sections, with the signal markedly reduced after preincubation with onalespib or the structurally unrelated inhibitor HSP990. Cell binding assays further confirmed saturable uptake in all three cancer cell lines, with substantial inhibition in the presence of onalespib and other Hsp90 inhibitors. *Ex vivo* biodistribution studies revealed pronounced abdominal activity and moderate uptake in U‑87 MG xenografts, whereas MDA‑MB‑231 tumours showed only minimal uptake, both with negligible brain signal under the same conditions. PET/CT imaging in both tumour models corroborated these findings and demonstrated effective *in vivo* blocking by HSP990 in U‑87 MG xenografts, together with limited penetration of [^11^C]Onalespib into the brain. Plasma metabolite analysis in healthy mice indicated rapid metabolic degradation, with only a small fraction of intact tracer remaining at all measured time points, despite the good *in vitro* stability in phosphate buffer, mouse serum and human serum.

**Conclusions:**

[^11^C]Onalespib was obtained with high purity and showed Hsp90-specific binding in tumour tissues, cells and brain tissue *in vitro*. *In vivo* PET/CT and biodistribution confirmed high abdominal accumulation, moderate tumour uptake and negligible brain penetration. Despite rapid metabolism, the tracer demonstrates clear Hsp90 specificity and requires further optimisation to develop a Hsp90-targeted radiotracers.

**Clinical trial number:**

not applicable

## Background

Heat shock protein 90 (Hsp90) is a molecular chaperone that plays a crucial role in maintaining essential cellular processes such as proteostasis and cell signalling. Structurally, Hsp90 is a homodimer where each monomer consists of three major domains: the N-terminal domain harbouring an adenosine triphosphate (ATP) binding site, the middle domain where most of the client protein interactions occur, and the C-terminal dimerization domain. In mammals, four distinct Hsp90 isoforms can be found, with Hsp90α and Hsp90β located predominantly in the cytosol, glucose-regulated protein 94 (GRP94) in the endoplasmic reticulum (ER), and tumour necrosis factor receptor-associated protein 1 (TRAP1) in the mitochondria. Hsp90 interacts with hundreds of client proteins, ensuring their folding, disaggregation, and degradation. Importantly, Hsp90 has been identified as a key contributor to tumourigenesis, supporting cancer cell survival and invasion through stabilization and activation of a plethora of oncogenic client proteins such as human epidermal growth factor receptor (HER2), Akt, and mutant p53 (Basso et al. 2002; Müller et al. 2005; Zsebik et al. 2006). Hsp90 inhibition leads to the destabilization and degradation of its oncogenic clients, thereby representing a potent tumour-targeting strategy.

Reductions in client protein levels following Hsp90 inhibition have been consistently documented in both preclinical and clinical studies. For instance, in a phase I/II trial, decreased uptake of the HER2-targeting radiotracer [^89^Zr]Zr-trastuzumab correlated with tumour shrinkage upon treatment with the Hsp90 inhibitor luminespib, also known as NVP-AUY922 (Gaykema et al. 2014). Furthermore, elevated Hsp90 expression has been reported across multiple tumour types compared to the healthy tissues. Increased serum levels of Hsp90 in hepatocellular carcinoma and ovarian cancer patients were associated with poor prognosis, suggesting its potential utility as a prognostic biomarker (Duan et al. 2023; Wei et al. 2020). High expression of cytosolic Hsp90α and Hsp90β in primary colorectal cancer patients correlated positively with poor survival, and can serve as a predictor of response to Hsp90-targeting therapies (Schmitz et al. 2025).

Over the past decades, numerous clinical trials have investigated Hsp90 inhibitors for cancer therapy, culminating in the approval of pimitespib (formerly known as TAS-116 and marketed as Jeselhy^®^) in 2022 for the treatment of gastrointestinal stromal tumours, with additional indications currently under investigation (Hoy 2022; Kurokawa et al. 2022). The growing interest in Hsp90 as a therapeutic target in cancer underscores the need for reliable tools to non-invasively assess Hsp90 expression in tumours. Such tools would facilitate patient selection for clinical trials, therapy, treatment monitoring and disease prognosis. Positron emission tomography (PET) and single-photon emission computed tomography (SPECT) imaging of Hsp90-overexpressing tumours have been demonstrated in preclinical and clinical studies. However, most of the currently developed probes exhibit limitations, including low tumour uptake, rapid metabolism, or unfavourable pharmacokinetics with high non-target organ accumulation, as summarized in our recent review (Cools et al. 2025b).

In addition to tumourigenesis, Hsp90 is also linked to the progression of neurodegenerative diseases such as Alzheimer’s disease (AD) and Parkinson’s disease (PD) (Chaudhury et al. 2021). Meta-analysis of the proteomics data of healthy and AD patients revealed downregulation of Hsp90 in the diseased brains (Koopman and Rüdiger 2020). Similarly, we observed a reduction of [^11^C]HSP990 tracer uptake in the major brain regions of aged and AD mice relative to the healthy young mice, suggesting the potential of Hsp90 PET as a diagnostic tool (Cools et al. 2025d).

**Fig. 1:**
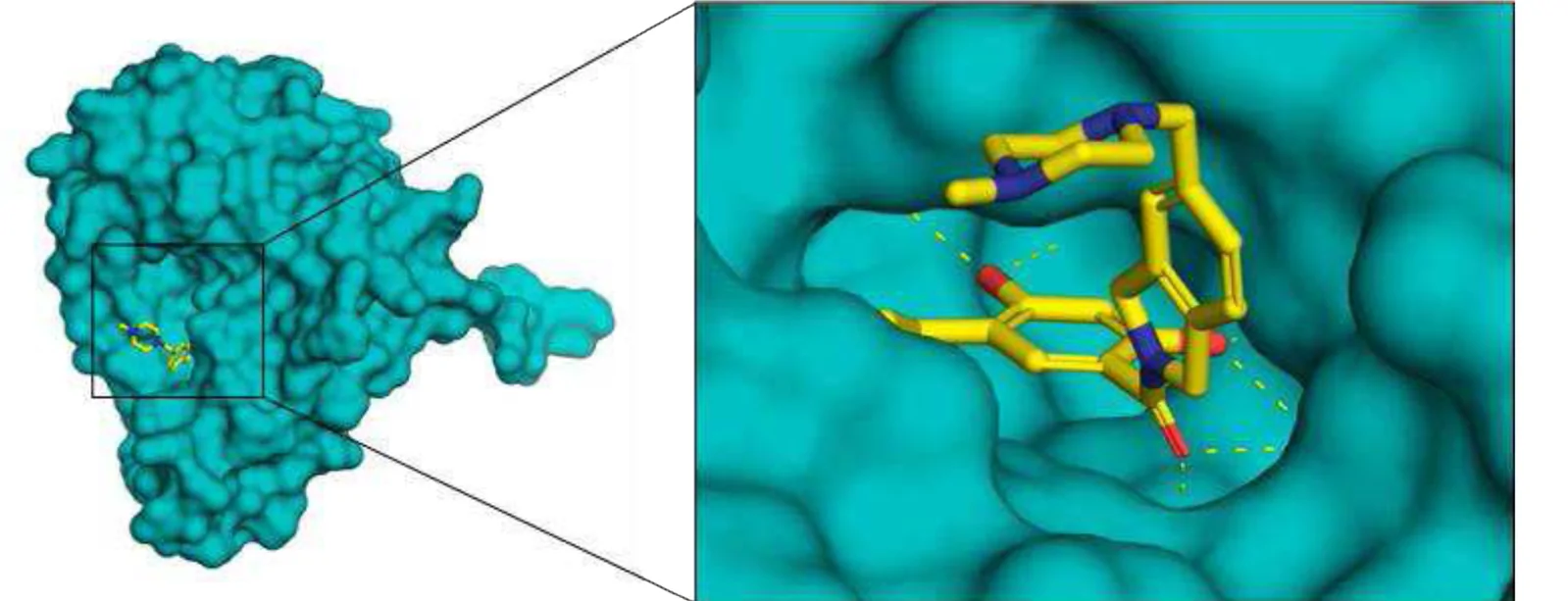
Structure of onalespib (shown in yellow, PDB ID 2XJX) bound to the N-terminal domain of Hsp90 (in turquoise). *N*-methylpiperazine moiety of onalespib protrudes outwards from the ATP-binding pocket of Hsp90 and is not involved in binding to Hsp90 (Woodhead et al. 2010).

Onalespib is a small molecule Hsp90 inhibitor that binds to the ATP pocket of Hsp90 and inhibits its function as a chaperone, similar to most of the developed Hsp90 drugs including HSP990 and pimitespib. 3 Preclinical studies have demonstrated its efficacy as monotherapy or in combination with chemo- and radiotherapy for cancer treatment, including in glioblastoma models (Canella et al. 2017; Spiegelberg et al. 2020; Uffenorde et al. 2025; Woodhead et al. 2010). Since its development in 2010, onalespib has been investigated in 13 Phase I and II clinical trials according to ClinicalTrials.gov. Its chemical structure offers an attractive opportunity: the piperazine moiety, located outside the ATP-binding pocket, can be potentially modified without disrupting Hsp90 binding (Fig. 1). Onalespib‑based derivatives have already been successfully applied in colorectal and gastric cancer models, where tumour uptakes of 10% ID/g and 5% ID/g were reported. Nevertheless, the onalespib scaffold itself has not yet been explored as a PET/SPECT imaging agent (Kennedy et al. 2026; Li et al. 2026). The current study aims to evaluate [^11^C]Onalespib as a PET probe for cancer and brain imaging through *in vitro* and *in vivo* studies, including assessment of Hsp90‑specific binding in tissues and cells, biodistribution, metabolic stability, and tumour and brain 14 accumulation in small rodents to provide the basis for its further development.

## Methods

### Reagents and chemicals

The reference compound onalespib, (2,4-dihydroxy-5-isopropylphenyl)(5-((4-methylpiperazin-1-yl)methyl)isoindolin-2-yl)methanone, other Hsp90 inhibitors, including HSP990 and pimitespib, were purchased from commercial suppliers (Selleckchem Chemicals or MedChem Express) and used without further purification (**Supplementary Fig. 1**, **Supplementary Table 1**). The precursor compound **4**, (2,4-dihydroxy-5-isopropylphenyl)(5-(piperazin-1-ylmethyl)isoindolin-2-yl)methanone, was synthesized following previously reported methods (Jiang et al. 2023) and the patented procedure (CN116947827A). A detailed synthetic protocol of intermediates compounds **1–4** and characterization is provided in the Supporting Information (**Supplementary Fig. 2****-****7**). Chemical structures were drawn using ChemDraw Ultra 15.0 (PerkinElmer).

### Radiosynthesis of [^11^C]Onalespib

Carbon-11 was produced *via* proton irradiation of a nitrogen/hydrogen gas mixture (N_2_ + 5% H_2_) using a Cyclone 18/9 cyclotron (IBA, Louvain-la-Neuve, Belgium). The radionuclide was obtained as [^11^C]CH^4^ through the nuclear reaction 1^4^N(p,α)^11^C. A custom-built gas-phase recirculation module was employed to convert [^11^C]CH^4^ into [^11^C]CH_3_I. The radiosynthesis of [^11^C]Onalespib was carried out *via N-*methylation by passing [^11^C]CH_3_I, carried by a helium stream, through a solution containing the onalespib precursor **4** (400 g) and *N*,*N*-diisopropylethylamine (DIPEA, 2–3 mg) dissolved in anhydrous dimethylsulfoxide (DMSO, 300 µL). The reaction was conducted at 100 °C for 4 minutes. Upon completion, the reaction mixture was cooled to 50 C and diluted with 1 mL of aqueous ammonium acetate solution (0.045 M, pH 6.9). Purification was performed by reversed-phase high-performance liquid chromatography (RP-HPLC) using an XBridge C_18_ column (5 µm, 4.6 × 150 mm, Waters, Milford, USA) and a mobile phase composed of ammonium acetate (0.045 M, pH 6.9) and ethanol (67:33, v/v) at a flow rate of 1.0 mL/min (**Supplementary Fig. 8**). The fraction containing [^11^C]Onalespib was collected and filtered into a sterile product vial using a single-use sterile syringe filter (Millex-GV, 0.22 µm, 13 mm diameter, Millipore, Billerica, MA). Subsequently, a 0.9% NaCl solution for injection was passed through the same filter into the vial to adjust the final ethanol concentration to below 8%. For quality control (QC), a 100 L aliquot was analysed by analytical HPLC. Chemical and radiochemical purity (RCP) were determined by RP-HPLC (BDS Hypersil C_18_ column, 5 µm, 100 × 3 mm, Thermo Fisher Scientific, Waltham, MA, USA) using a mobile phase consisting of 78% ammonium acetate (0.045 M, pH 6.9) and 22% CH_3_CN, at a flow rate of 1.20 mL/min. Tracer identity was confirmed by comparison with the non-radioactive reference compound under identical chromatographic conditions (**Supplementary Fig. 9**).

### *In vitro* tissue autoradiography

Autoradiography was performed as previously described (Cools et al. 2025a). Tissue samples were obtained from previous studies. Frozen tissue sections were thawed, air-dried and washed in tris(hydroxymethyl)aminomethane hydrochloride (Tris-HCl buffer, 50 mM, pH 7.4) for 10 min at room temperature (RT). Sections were air-dried and pre-incubated with either vehicle (Tris-HCl buffer, 50 mM, pH 7.4 containing 0.3% BSA + 1% DMSO) as baseline control or 10 µM of onalespib, HSP990, or pimitespib dissolved in vehicle for 10 min at RT. Next, blocking solutions were removed, sections were dipped in ice-cold Tris-HCl buffer (50 mM, pH 7.4) + 0.3% BSA and air-dried. Slices were incubated with [^11^C]Onalespib tracer (29–43 kBq/slide) in Tris-HCl buffer (50 mM, pH 7.4) + 0.3% BSA for 10 min at RT. Tracer solution was removed and the sections were washed twice for 5 min in ice-cold Tris-HCl buffer (50 mM, pH 7.4) + 0.3% BSA followed by a dip in ice-cold water. The air-dried tissue slices were exposed overnight to a phosphor storage screen (super-resolution screen, PerkinElmer, Waltham, MA) and the autoradiograms were obtained by reading the screens using a Cyclone Plus system (PerkinElmer). Autoradiography images were analysed using OptiQuant software (PerkinElmer), and intensity was expressed in digital light units per mm squared (DLU/mm^2^). Percent blocking was calculated as (1 – ((average DLU/mm^2^ in the presence of 10 µM blocker) / (average DLU/mm^2^ tracer only))) 100, and data were presented as mean – standard deviation (SD), N = 3 tissue sections per pre-incubation condition.

### Cell culture

MDA-MB-231, U-87 MG, and PC-3 cell lines were obtained from American Type Culture Collection (ATCC) and grown in Dulbecco’s Modified Eagle’s Medium (DMEM, high glucose, Gibco 500 mL, Fischer Scientific: 41965–039) supplemented with 10% fetal bovine serum (FBS), 1X MEM non-essential amino acids (Gibco, Fischer Scientific: 11140–035) or 1 mM Sodium Pyruvate (Gibco, Fischer Scientific: 11360–039). Cells were kept in the humidified incubator at 37 °C and 5% CO_2_ and subcultured using TrypLE^™^ Select Enzyme (1X), no phenol red, (Gibco, Fischer Scientific: 12563029). Cell passage was kept under 20 for all experiments performed in this study. All cells were kept free of mycoplasma contamination throughout all studies.

### *In vitro* cell binding

Cell binding experiments were performed as previously described (Cools et al. 2025a). Briefly, cells were seeded at 100,000–125,000 cells/well density in 6-well plates and incubated at 37 °C for 48 h in the presence of 5% CO_2_. On the day of the cell binding, cells were gently washed once with phosphate-buffered saline (PBS) and preincubated with either vehicle (cell culture medium + 1% DMSO) as baseline control or 100 µM of onalespib, HSP990, or pimitespib dissolved in vehicle for 60 min at 37 °C and 5% CO_2_. Next, the preincubation solutions were removed, and the cells were gently washed once with PBS. The cells were subsequently incubated with 555 kBq/well of [^11^C]Onalespib dissolved in cell culture medium (total 1% ethanol) for 30 min at 37 °C. Cells were collected and activity was measured in a gamma counter, followed by cell number quantification using an automated cell counter NucleoCounter^®^ NC-100^™^ (Chemometec). Data were expressed as percentage of the applied radioactivity bound to 1 × 10^5^ cells normalized to the baseline control conditions and presented as mean ± SD, N = 3 wells per pre-incubation condition, using GraphPad Prism 10.0 software.

### Animals

All mice were kept in a thermoregulated (22 C) and humidity-controlled environment with a 12 h light / 12 h dark cycle in individual ventilated cages and had free access to food and water. All animal experiments were conducted after approval of the local University Ethics Committee for Animals (023/2025) and carried out in accordance with the ARRIVE guidelines. Female CB17.Cg-*Prkdc*^*scid*^*Lyst*^*bgJ*^/Crl (SCID/beige) mice, 5–6 weeks old (body weight 15–20 g), were purchased from Charles River Laboratories (Sulzfeld, Germany). Female NMRI mice, 10–15 weeks old (body weight 25–35 g), were obtained from Inotiv (Horst, the Netherlands). Female animals were chosen as they are generally more docile in nature and do not grow as large as male mice.

### Quantification of radioactivity in biological samples

Quantification of radioactivity was performed with an automated gamma counter equipped with a 3-inch NaI(Tl) well crystal coupled to a multichannel analyser (Wallac 1480 Wizard, Wallac, Turku, Finland). The results were corrected for background radiation, physical decay during counting and detector dead time.

### *In vitro* serum stability

PBS solution, mouse and human serum (400 µL) were pre-warmed to 37 °C for 5 min. Subsequently, 10 µL of [^11^C]Onalespib (~ 0.5 MBq) was added, and the samples were incubated at 37 °C for 30 min. At 10 and 30 min, aliquots of 100 µL were withdrawn and proteins were denatured by adding 100 µL of ice-cold acetonitrile, followed by vortexing for 10 s and centrifugation at 4000 rpm for 5 min. The fraction of intact tracer in the supernatant was quantified by RP-HPLC using a Chromolith RP C_18_ column (3 mm × 100 mm, Merck, Darmstadt, Germany) with the following gradient conditions: 0–2 min, 1% B; 2–2.1 min, 1% to 15% B; 2.1–12 min, 15% to 35% B; 12–12.1 min, 35% to 95% B; 12.1–15 min, 95% B. A = 0.05M sodium acetate buffer (pH 5.5) and B = CH_3_CN. HPLC analysis were carried out on a LaChrom Elite system (Hitachi, Darmstadt, Germany) equipped with a Waters 2487 UV-Vis detector set at 254 nm and a 3-inch NaI(Tl) scintillation detector connected to a single-channel analyser (Gabi, Raytest, Straubenhardt, Germany). Chromatogram acquisition and integration were performed using GINA Star (Raytest) or RaChel (Lablogic, Sheffield, UK) software. Data were presented using GraphPad Prism 10.0 software.

### Plasma radiometabolites study

The plasma radiometabolites study was performed as previously described (Vermeulen et al. 2019). Healthy female NMRI mice were anesthetized with 1.5–2.5% isoflurane in oxygen (flow rate 1.0 L/min) and injected intravenously (*i.v.*) *via* a tail vein with approximately 4–10 MBq of the tracer. Animals were sacrificed by decapitation at 10- and 30-min post-injection (*p.i.*), N = 4 per time point. Blood was collected into K_2_EDTA-coated tubes (BD Vacutainer, BD, Franklin Lakes, NJ, USA) and kept on ice. Plasma was obtained by centrifugation for 5 min at 2330 x g, and individual plasma samples (50 µL) were subsequently analysed by RP-HPLC under the same chromatographic conditions as those used for the *in vitro* serum stability study.

### Generation of tumour models

Mice were anesthetized with 1.5–2.5% isoflurane in O_2_ at a flow rate of 1.0 L/min. A total of 1 × 10^6^ MDA-MB-231 or U-87 MG cells, mixed with Cultrex (1:1; Cultrex Basement Membrane Extract, R&D systems) to a final volume of 100 µL per mouse, were subcutaneously inoculated into the left shoulder of female 10-week-old SCID/beige mice. The tumours were allowed to grow for 4–8 weeks until they reached visible size ranging ~80–450 mm^3^ (measured by calliper, 0.5 × L × W^2^), after which the animals were used for *ex vivo* biodistribution and *in vivo* imaging studies.

### *Ex vivo* biodistribution

*Ex vivo* biodistribution studies were performed in SCID/beige mice bearing MDA-MB-231 and U-87 MG tumours. Mice were anesthetized using 1.5–2.5% isoflurane in O_2_ at a flow rate of 1.0 L/min and injected *i.v.* with ~5 MBq of [^11^C]Onalespib *via* a tail vein. The mice were subsequently sacrificed by decapitation at 30 min post-tracer injection. All organs of interest, including blood, were collected in pre-tared tubes and weighed. The radioactivity in each organ was counted in an automated gamma counter. For the calculation of the total radioactivity in blood, muscle and bone, the masses were assumed to be 7%, 40% and 12% of the total body mass, respectively (Burns et al. 2007; Horti et al. 2006; Vermeulen et al. 2019). Data were expressed as percentage of injected dose (%ID), calculated as (counts per min (CPM) in organ / total CPM recovered) × 100, and SUV calculated as (radioactivity in CPM in organ / weight of organ in g) / (total CPM recovered / total body weight in g). Data were presented using GraphPad Prism 10.0 software as mean ± SD, N = 4 per time point.

### *In vivo* [^11^C]Onalespib PET/CT imaging

MDA-MB-231 and U-87 MG tumour-bearing mice were imaged after injection of [^11^C]Onalespib, 4–5 weeks after cell inoculation. Mice were anesthetized using 1.5–2.5% isoflurane in O_2_ at a flow rate of 1.0 L/min, injected *i.v.* with ~ 4 MBq of [^11^C]Onalespib *via* a tail vein and scanned dynamically for 90 min (Molecubes β-CUBE), followed by a CT scan (Molecubes X-CUBE), allowing full body field of view PET/CT imaging. To assess Hsp90 binding specificity, mice were pre-treated by *i.p.* injection 60 min before injection of [^11^C]Onalespib with either vehicle (an aqueous solution of 10% DMSO and 90% H_2_O containing 40% of (2-hydroxypropyl)-β-cyclodextrin) as baseline control or HSP990 (5 mg/kg) dissolved in vehicle. Pre-treatment solutions were sterile-filtered through a 0.22 m membrane filter (Millex-GV, Millipore). Dynamic PET data were histogrammed into 24 frames (4 × 15 s, 4 × 1 min, 5 × 3 min, 8 × 5 min, 3 × 10 min) and reconstructed using 30 iterations of the manufacturer’s MLEM algorithm with corrections for randoms, scatter, attenuation and decay into a 192 × 192 image matrix containing 0.4 mm voxels. CT data were reconstructed using a regularized statistical (iterative) image reconstruction algorithm 2 using non-negative least squares, using an isotropic 200 m voxel size and scaled to Hounsfield Units after 3 calibration against a standard air/water phantom. Analysis of the PET/CT images was performed using the 4 PMOD version 4.4 software (PMOD Technologies Ltd., Zurich, Switzerland). Activity is expressed as standardized uptake value (SUV mean), maximum intensity projection (MIP) images were created from frames 22–24, N = 4 per pre-treatment condition.

## Results

### Radiosynthesis of [^11^C]Onalespib

**Fig. 2:**
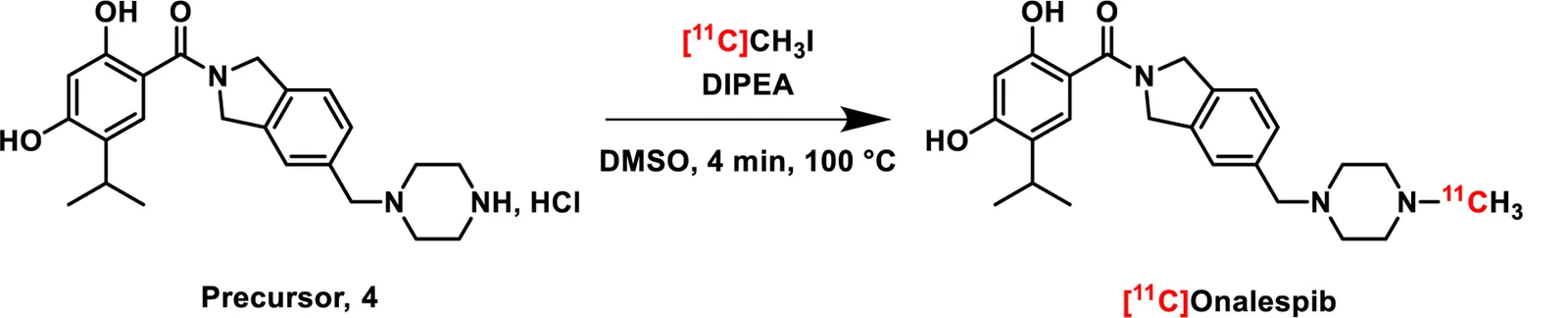
Radiosynthesis of [^11^C]Onalespib by *N*-methylation of precursor **4** with [^11^C]CH_3_I.

Precursor compound **4** was synthesized in four steps, affording an overall yield of 36% (**Supplementary Fig. 2**). [^11^C]Onalespib was obtained by *N*-methylation of the piperazine moiety of the precursor **4** (Fig. 2), resulting in a radiochemical conversion of 67 ± 8% (N = 12), as determined by integration of the area under curve of peaks observed on preparative HPLC (**Supplementary Fig. 8**). [^11^C]Onalespib was purified by RP-HPLC with a radiochemical purity of >99% and a molar activity of 30 ± 6 GBq/mol at the end of synthesis (N = 3). The QC chromatogram of [^11^C]Onalespib and the authentic reference compound onalespib showed identical retention times for both compounds, confirming the identity of the synthesized radiotracer (**Supplementary Fig. 9**). Additional non-radioactive experiments 17 were also performed to confirm methylation on the piperazine moiety (**Supplementary Fig. 10****-****11**).

### *In vitro* evaluation of [^11^C]Onalespib

**Fig. 3:**
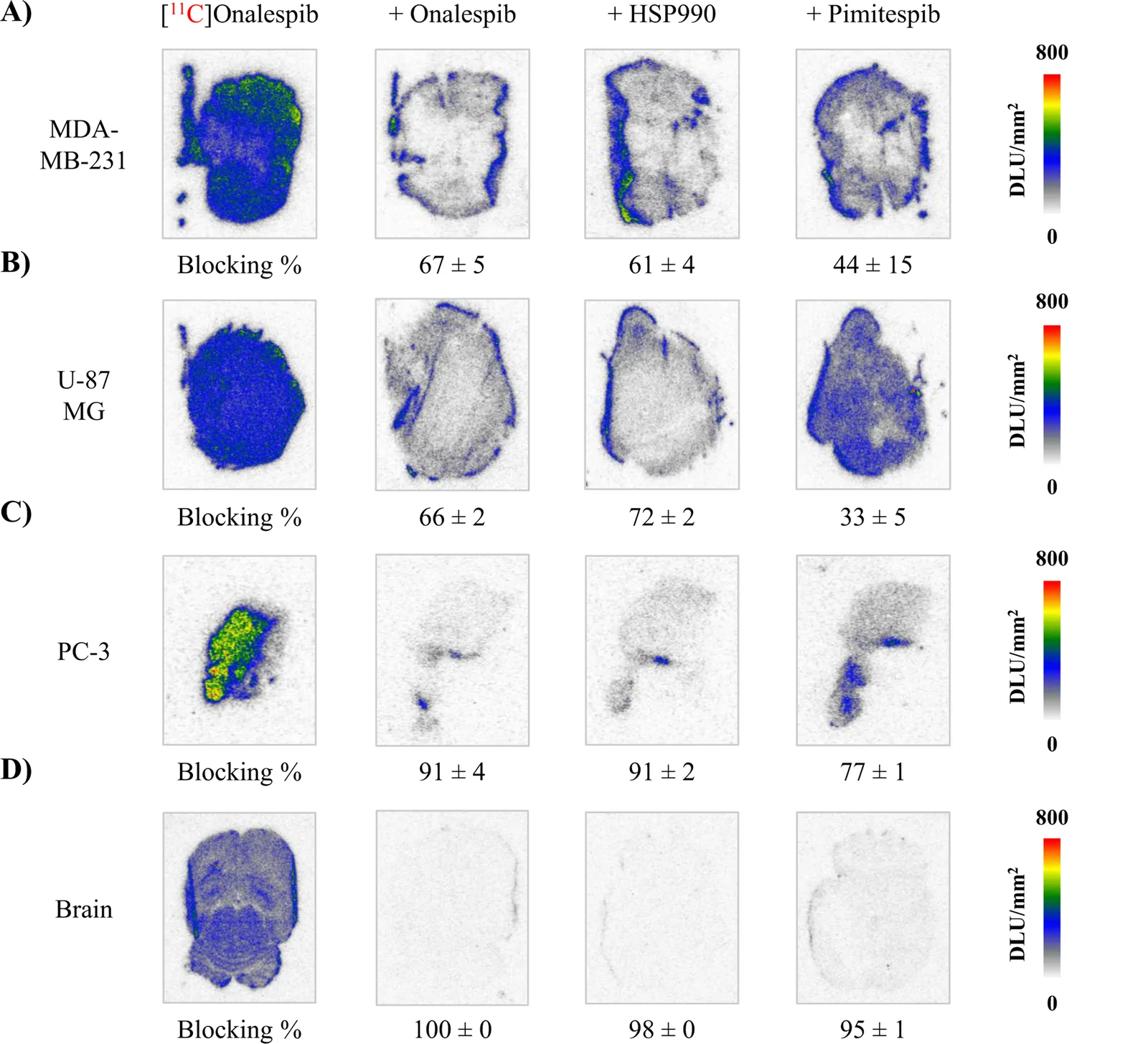
*In vitro* [^11^C]Onalespib autoradiography. Sections were pre-incubated with vehicle (baseline) or 10 µM Hsp90 inhibitors (block) followed by 23–43 kBq/slide [^11^C]Onalespib. **A**) MDA-MB-231 breast tumour, **B**) U-87 MG glioblastoma, **C)** PC-3 prostate tumour, and **D)** healthy rat brain. The blocking efficiency is shown for each condition and expressed as %blocking ± SD, N = 3. Intensity is expressed in dynamic light units per mm^2^ (DLU/mm^2^). A representative slide for each condition is shown.

*In vitro* autoradiography demonstrated high and Hsp90-specific binding of [^11^C]Onalespib to three tumour tissue type cryosections: triple negative breast cancer (MDA-MB-231), glioblastoma (U-87 MG), and prostate cancer (PC-3). Significant tracer binding was also observed to mouse brain cryosections (Fig. 3). Preincubation of tissue sections with 10 µM onalespib, HSP990 or pimitespib, two structurally unrelated Hsp90 inhibitors, markedly reduced tracer binding, confirming saturable and Hsp90-specific interactions. In PC-3 tumour tissue, tracer binding was effectively blocked by >90% with onalespib and HSP990, whereas pimitespib showed lower blocking efficiency across all tumour types. Brain tissue slices exhibited pronounced Hsp90-specific [^11^C]Onalespib binding, with blocking efficiencies exceeding 95% for all 10 inhibitors tested.

**Fig. 4:**
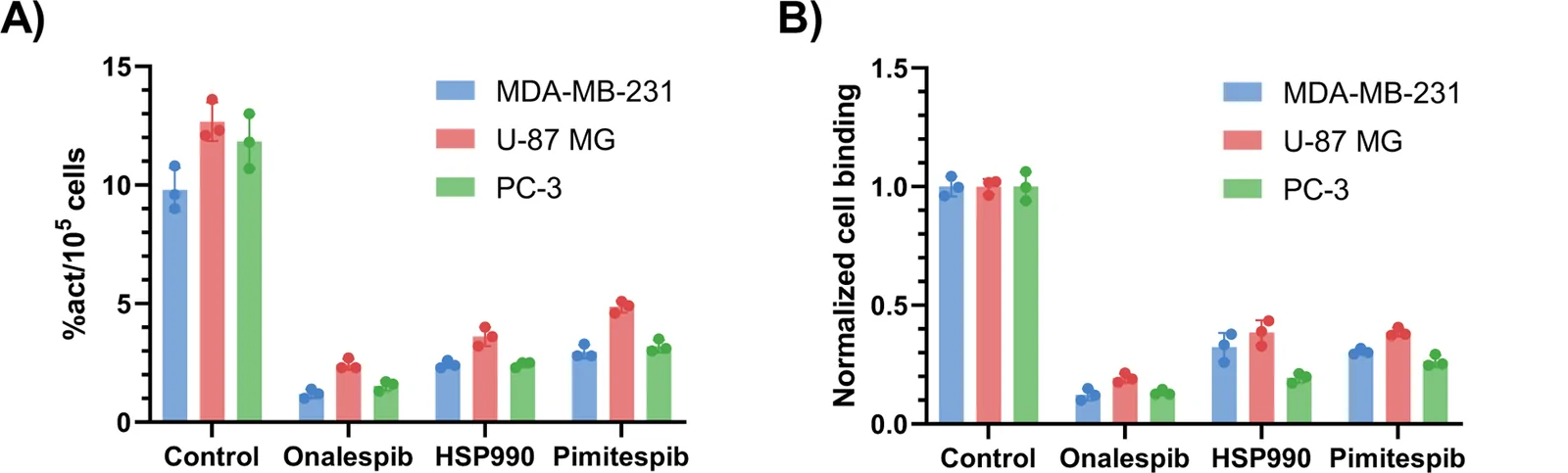
*In vitro* cell binding of [^11^C]Onalespib to U-87 MG, MDA-MB-231 and PC-3 cells treated with either DMSO vehicle or 100 µM of Hsp90 inhibitors onalespib, HSP990, or pimitespib for 1 hour prior to tracer incubation. **A)** Data are shown as % of applied radioactivity bound to 1 × 10^5^ cells (%act/10^5^ cells) **B)** Data are normalized to the baseline control conditions. Data in A-B are presented as mean ± SD, N = 3.

To investigate the binding of [^11^C]Onalespib in live cells, a cell-binding assay was performed on the same tumour cell lines used in the tissue autoradiography experiment (Fig. 4). Cellular tracer uptake was high in every cell line (~10–12% activity/10^5^ cells). Consistent with the observations in tumour tissues, saturable [^11^C]Onalespib uptake was also detected across the same cancer cells. Maximum blocking was achieved by co-incubation with onalespib, resulting in more than 80% reduction in tracer binding compared to DMSO vehicle control. In contrast, blocking with structurally unrelated inhibitors HSP990 and pimitespib moderately reduced tracer binding to 20–40% compared to DMSO vehicle control.

### *Ex vivo* biodistribution and *in vivo* PET/CT imaging in U-87 MG and MDA-MB-231 tumour-bearing mice

**Fig. 5:**
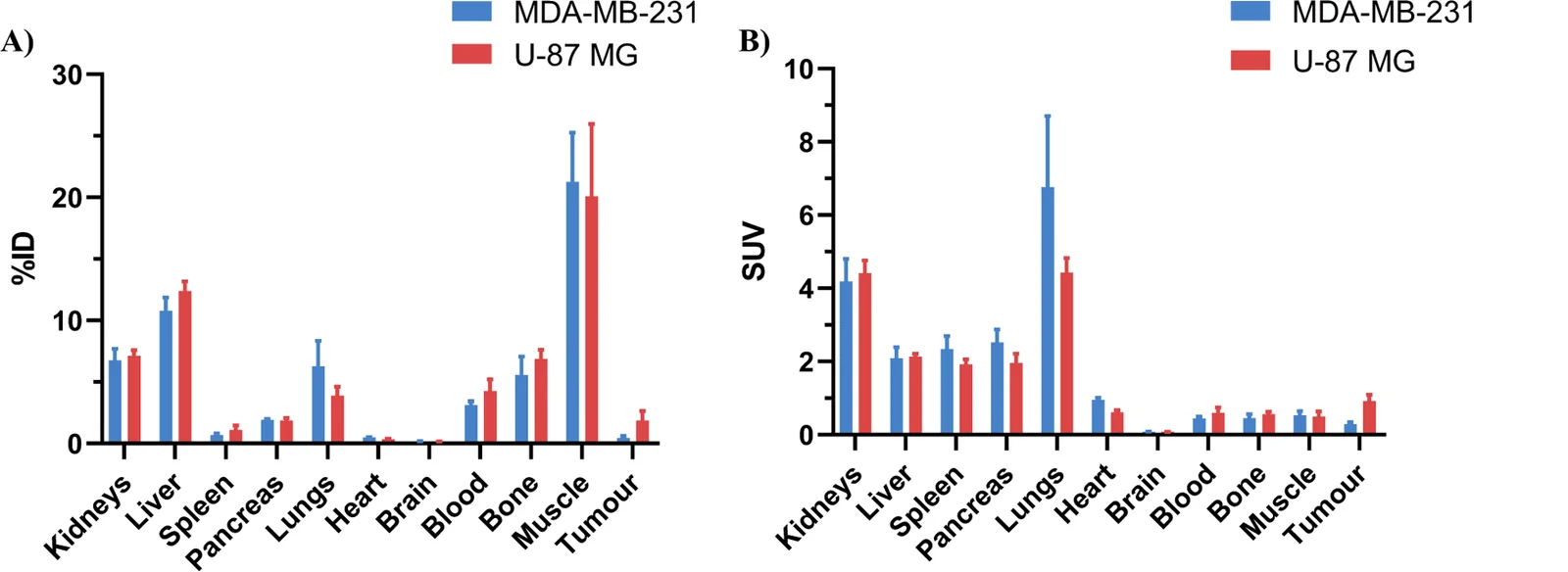
[^11^C]Onalespib biodistribution in MDA-MB-231 and U-87 MG tumour-bearing mice 30 min *p.i.* Tracer uptake is expressed as **A)** percent injected dose per gram tissue %ID and **B)** standardized uptake value SUV. Data are shown as mean ± SD, N=4.

*Ex vivo* biodistribution at 30 min *p.i*. in tumour-bearing mice showed high tracer uptake in kidneys, liver, spleen, pancreas, and lungs (Fig. 5, **Supplementary Table 2**). Tumour uptake was moderate in U-87 15 MG xenograft mice (4.9 ± 0.9 %ID/g, SUV 0.9 ± 0.2), whereas substantially lower uptake was observed in MDA-MB-231 xenografts (1.6 ± 0.3 %ID/g, SUV 0.3 ± 0.1). Brain uptake was negligible.

**Fig. 6:**
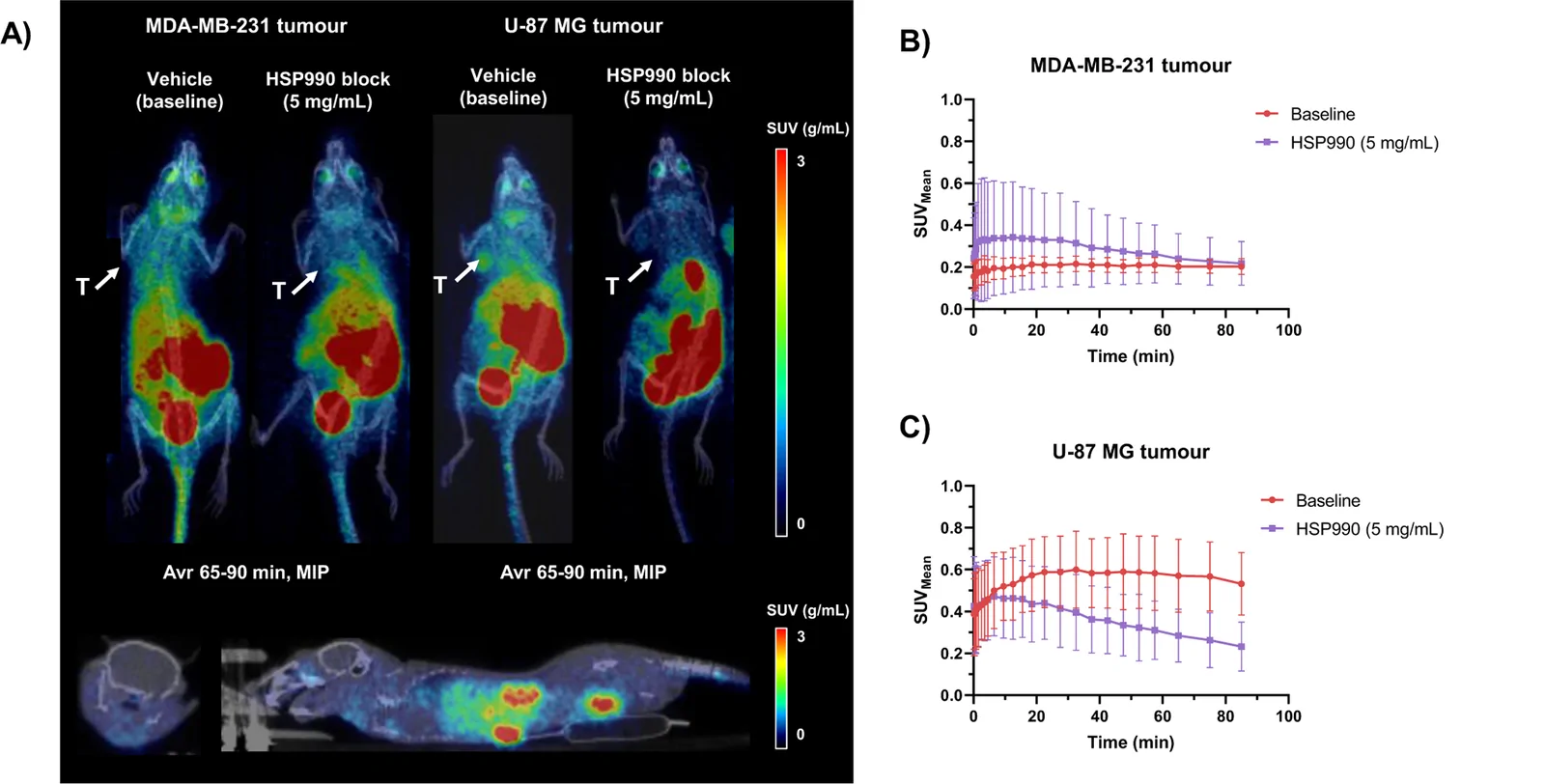
Maximum intensity projection (MIP) of [^11^C]Onalespib PET scan co-registered with CT, intensity is expressed in SUV (g/mL). **A)** Coronal view of baseline and HSP990 (5mg/mL) block in MDA-MB-231 (left) and U-87 MG (right) tumour-bearing mice; in the baseline scan in sagittal (bottom left) and transaxial (bottom right) view. A representative image for all mice is shown. Time-activity curves of tumour uptake in **B)** MDA-MB-231 and **C)** U-87 MG xenografts are shown for block *vs* baseline conditions. Data are presented as mean ± SD, N=4.

Similar to *ex vivo* data, moderate tumour uptake of [^11^C]Onalespib was observed in U-87 MG, but not in MDA-MB-231 xenograft mice in PET/CT. [^11^C]Onalespib uptake in U-87 MG tumours was blocked by pre-treatment with structurally unrelated inhibitor, HSP990 (5 mg/kg), administered *i.p.* 60 min before tracer injection (Fig. 6). Similar to *ex vivo* results and comparable to the previously reported small molecule Hsp90 tracers, high uptake in abdominal region can be observed, with kidneys and GI tract clearly visible in the images. No brain uptake of [^11^C]Onalespib was detected by PET/CT imaging, consistent with *ex vivo* biodistribution data. Although some signal appears close to the brain region in the coronal view, the sagittal and transaxial views confirm that there is no activity within the brain itself.

### *In vitro* serum stability and *in vivo* plasma radiometabolites

**Fig. 7:**
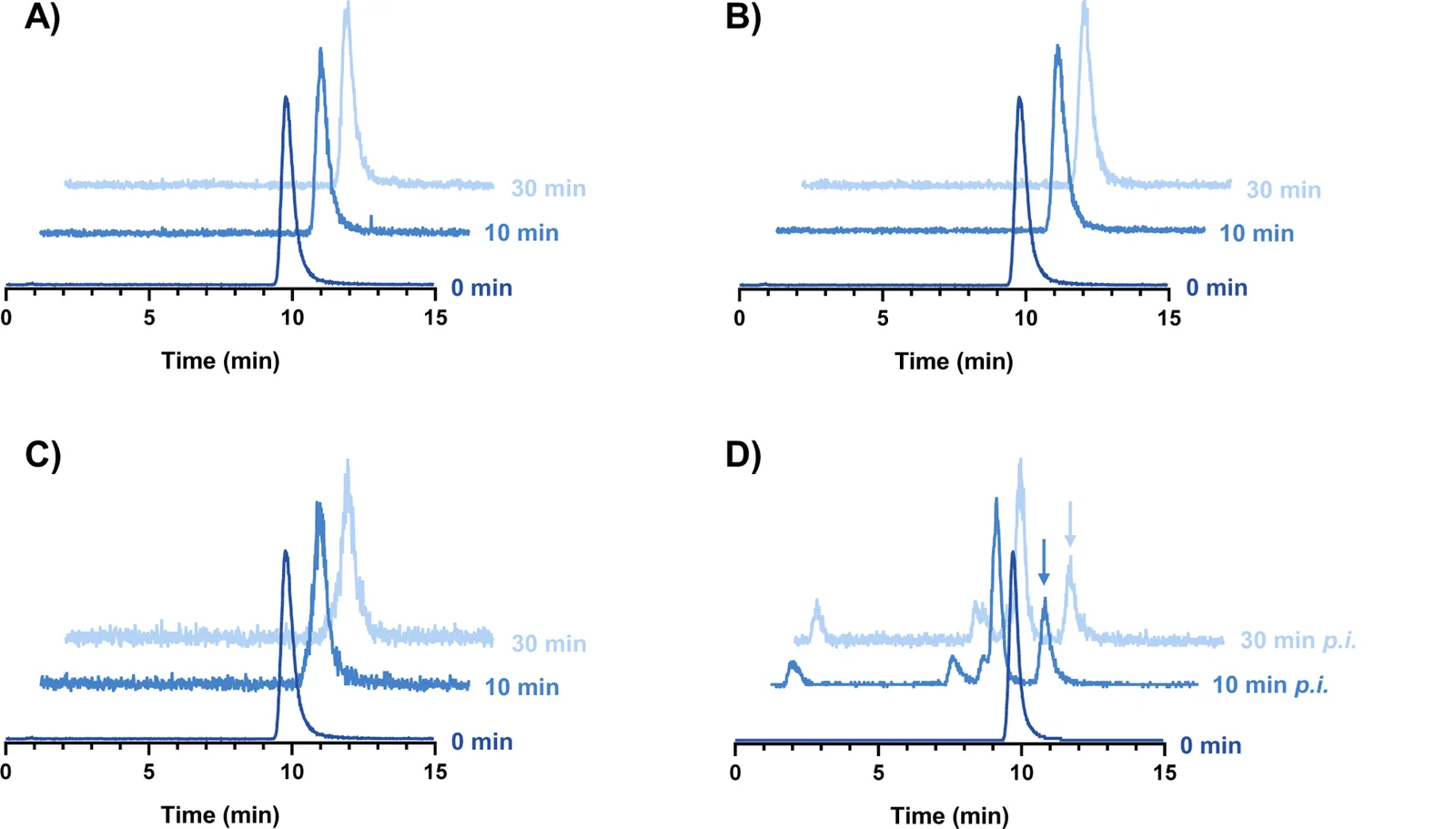
Radio-HPLC chromatograms showing the stability of [^11^C]Onalespib after incubation for 10 and 30 min in **A)** PBS solution, **B)** mouse serum, **C)** human serum. **D)** In vivo metabolic stability of [^11^C]Onalespib in mouse plasma collected at 10 and 30 min *p.i*.. N = 4 per time point.

*In vitro* stability studies indicated that [^11^C]Onalespib is stable after incubation in PBS buffer, mice and human serum at 37 °C for 30 min (Fig. 7A-C). However, an *in vivo* plasma radiometabolite study in mice revealed rapid metabolism of [^11^C]Onalespib, with 21 ± 4% of intact tracer remaining after 10 minutes *p.i.* and 16 ± 8% after 30 minutes. At all examined time points, the detected radiometabolites were more polar than the parent [^11^C]Onalespib compound (Fig. 7D).

## Discussion

Hsp90 remains an attractive target for cancer treatment given its entanglement in multiple cancer hallmarks, with Hsp90 inhibitors showing promise across a wide range of tumour types in preclinical and clinical studies. In addition to the standalone treatment with Hsp90 inhibitors, multiple combination therapies are explored including chemo-, immuno-, and radiotherapies (Ren et al. 2022). Interestingly, multiple Hsp90 inhibitors, including onalespib, showed promising preclinical results as a radiosensitizers of glioblastoma, a very aggressive tumour type with poor survival (Canella et al. 2017). Given the central role of Hsp90 in cancer biology, this study aimed to develop and evaluate [^11^C]Onalespib as a novel PET tracer for Hsp90 imaging.

The selection of onalespib for the development of an Hsp90‑targeted PET tracer was driven by the observation that the piperazine moiety points outwards from the ATP‑binding pocket of Hsp90, and is thus not involved in Hsp90 binding (Woodhead et al. 2010). This structural feature may allow optimization of tracer pharmacokinetics and incorporation of chelator/radiometals, where longer half-life as well as its therapeutic potential with α- and β^-^-emitters can be explored. Such modifications could overcome limitations observed with our previously developed radiotracer [^11^C]HSP990, for which the introduction of a fluoroethyl group to generate the fluorine-18 analogue [^18^F]FEHSP990 resulted in loss of affinity for Hsp90 (Cools et al. 2025a; Cools et al. 2025c). Emerging interest in onalespib‑based imaging agents has highlighted the potential of targeting the piperazine moiety for radiotracer development. Recently, onalespib has been successfully derivatized at the piperazine moiety to incorporate hydrazinonicotinamide and DOTA chelators, enabling SPECT imaging with technetium‑99m and PET imaging with gallium‑68, respectively (Li et al. 2026). This strategy yielded promising radiotracers for Hsp90 imaging and supports further development of onalespib‑based agents. In parallel, an other study examined structural modifications on both the piperazine and isopropyl groups of onalespib. Substitution of the isopropyl group with bromine or iodine was tolerated but reduced affinity for Hsp90, with the larger iodine atom causing a more pronounced loss. Replacement of the piperazine ring with a stereochemically defined piperidine restored the affinity of the brominated analogue. Notably, direct derivatization of the piperazine moiety without altering the remainder of the scaffold has not yet been explored (Kennedy et al. 2026).

### [^11^C]Onalespib demonstrates Hsp90-specific binding *in vitro* and in U-87 MG xenograft

We found that [^11^C]Onalespib binds specifically to Hsp90 in various tumour and brain tissues, as demonstrated by the blocking studies using structurally unrelated Hsp90 inhibitors, including the clinically approved inhibitor pimitespib. While strong blocking was observed in PC‑3 tumour and brain tissues, heterogeneous tracer distribution was observed in MDA‑MB‑231 and U‑87 MG sections. In these tissues, the peripheral regions of the slices exhibited the highest tracer binding levels, leading to the incomplete blocking of [^11^C]Onalespib, particularly when pimitespib was used as a blocking agent. Tissue thickness is often higher around the edges due to tissue folding, which can hamper efficient removal of unbound tracer during the washing steps. However, even when border regions were excluded from the analysis, blocking efficiency remained similar and still incomplete (**Supplementary Fig. 12****–****13**). Incomplete blocking may signal non‑specific binding of the tracer, as well as differential selectivity of the tested compounds toward the various Hsp90 isoforms. Onalespib is designated as a pan‑Hsp90 inhibitor and shows minimal isoform selectivity, with comparable affinities for Hsp90α (Kd = 0.05 μM) and GRP94 (Kd = 0.03 μM) (Zhang et al. 2025). In contrast, HSP990 and pimitespib preferentially target the cytosolic isoforms Hsp90α and Hsp90β with high affinity in the low‑nanomolar range (**Supplementary Table 1**). As pimitespib displays both higher isoform selectivity and lower affinity for Hsp90, a fraction of [^11^C]Onalespib remains bound to Hsp90 sites that are not fully saturated by the inhibitor, which likely accounts for the incomplete blocking observed. Similar results have been observed on cultured cells, particularly when non‑homologous Hsp90 inhibitors were used. Given the lack of complete blocking of the [^11^C]Onalespib uptake in these experiments, Hsp90 specificity could be further investigated using isoform-specific inhibitors and cell models with knockdown and/or knockout of individual Hsp90 isoforms, including GRP94 and TRAP1. Another possible explanation could be off-target (non-Hsp90) binding of the tracer. While onalespib did not display any off-target kinase pharmacology in a 16-kinase panel (Antolin et al. 2021), it might still engage with targets other than Hsp90, explaining incomplete self-blocking.

Tracer accumulation in U-87 MG xenograft was detected and was higher under baseline conditions compared to blocking with the non-homologous inhibitor HSP990, suggesting Hsp90 binding specificity *in vivo*. Tumour uptake was comparable to most other Hsp90 radiotracers such as [^11^C]NMS-E973 and [^11^C]YC-72-AB85 with SUV ~1 in B16.F10 melanoma model, whereas [^11^C]HSP990 had a slightly higher uptake of ~2 SUV in the same U-87 MG tumour model as used in the current study (Cools et al. 2025a; Vermeulen et al. 2021; Vermeulen et al. 2019). Similar to our previous studies with [^11^C]HSP990, no tumour uptake was observed in MDA-MB-231, likely due to the differential expression of Hsp90 *in vitro vs. in vivo* (Cools et al. 2025a).

### Biodistribution of [^11^C]Onalespib is comparable to the previously developed Hsp90 tracers

[^11^C]Onalespib *ex vivo* and *in vivo* biodistribution did not dramatically differ from the other small molecule Hsp90 radiotracers reported so far, such as [^11^C]NMS-E973, [^11^C]YC-72-AB85, and [^11^C]HSP990 (Cools et al. 2025a; Vermeulen et al. 2021; Vermeulen et al. 2019). Compared to our most advanced [^11^C]HSP990 radiotracer, [^11^C]Onalespib shows higher renal/urinary excretion, with a two-fold higher SUV observed in kidneys at 30 min *p.i.* However, hepatobiliary clearance remains the major route of tracer elimination. Additionally, high activity was detected in the lungs and was several folds higher than in the heart, suggesting tracer retention in the organ *vs.* result of blood circulation. Derivatization of onalespib on the isopropyl and piperazine group without a significant loss of affinity has been previously demonstrated (Kennedy et al. 2026). Recently reported onalespib derivatives [^99m^Tc]Tc-YQHSP-02 and [^68^Ga]Ga-YQHSP-02 with an optimized linker connecting Hsp90 inhibitor to Technetium-99m or Gallium-68 chelators, respectively, showed enhanced biodistribution profile (Li et al. 2026). Renal clearance was observed as a major elimination route of [^99m^Tc]Tc-YQHSP-02, and high tumour uptake (9% ID/g) was observed as early as 30 min *p.i.* in HCT116 tumour-bearing mice. Last generation Sansalvamide peptide radiotracer, [^18^F]PEGylated San A, showed a similar tumour accumulation in PL45 pancreatic cancer with 5.22 ± 1.94 %ID/g, which is only slightly higher than that of [^11^C]Onalespib with 4.9 ± 0.9 %ID/g in U-87 MG, although activity in the non-target organs was considerably lower for the peptide tracer (Wang et al. 2024).

### [^11^C]Onalespib exhibits poor brain uptake *in vivo*

In the study investigating onalespib as a radiosensitiser of glioblastoma, blood-brain barrier (BBB) permeability of onalespib was assessed by LC-MS/MS analysis of brain tissue after administering the drug *via* tail vein injection. Higher levels of onalespib were observed in the brain relative to the plasma levels at 2–24 hours post-administration, suggesting its BBB permeability (Canella et al. 2017). Surprisingly, our previous study of [^11^C]HSP990 radiotracer showed that treatment with onalespib did not block the brain uptake of [^11^C]HSP990, suggesting limited BBB permeability of onalespib contrary to the reported data (Cools et al. 2025a). Similarly, no brain uptake of onalespib was observed in our previous study when brain extracts were assayed by LC−MS/MS following *i.v.* injection of the inhibitor in healthy mice (Vermeulen et al. 2021). Together, these observations raise concerns whether onalespib’s use for the glioblastoma radiosensitization is a rational choice. In the current study, high Hsp90-specific binding of [^11^C]Onalespib to the brain tissue and to U-87 MG glioblastoma cells *in vitro* was observed. However, neither PET imaging over 90 min *p.i.*, nor *ex vivo* biodistribution at 30 min *p.i.* could confirm substantial brain uptake of [^11^C]Onalespib. These findings question the potential use of onalespib for glioblastoma application. It might be argued that the BBB in glioblastoma patients is disrupted, and therefore brain permeability of the drug is of less importance than the target engagement. However, Sarkaria et. al. highlight in their review that there is a significant fraction of tumour burden with intact BBB, and drugs that cannot cross it would be less effective for treatment of the disease (Sarkaria et al. 2018). For this reason, Hsp90 inhibitors with demonstrated brain uptake *in vivo* such as BIIB021 and HSP990 and their corresponding radiotracers [^11^C]BIIB021 and [^11^C]HSP990 might offer a better alternative to onalespib for sensitizing glioblastoma to radiotherapy and for imaging applications (Cools et al. 2025d; Sakai et al. 2024).

### Rapid metabolism of [^11^C]Onalespib was observed in healthy mice

Fast plasma clearance is advantageous for PET imaging when accompanied by rapid saturable tumour uptake and retention, providing a good image contrast. The presence of radiometabolites, however, can complicate quantitative image analysis as the PET scanner cannot differentiate between intact tracer and radiometabolites. Despite good *in vitro* stability of [^11^C]Onalespib in both buffer and mouse/human serum, a significant fraction of radiometabolites (79%) was present in plasma as soon as 10 min *p.i*. Rapid metabolism of onalespib has already been reported in both preclinical and clinical settings, driven by the formation of glucuronide and sulphate conjugates, likely responsible for the partial renal clearance of the drug (Shapiro et al. 2015; Woodhead et al. 2010). While the chemical identity of the radiometabolites was not established in the present study, glucuronide and sulphate adducts are some of the likely suspects as both would be more polar compared to the parent radiotracer. Although their presence can complicate image analysis, a substantial metabolic fraction also reduces the amount of intact tracer available for tumour uptake. Although this effect may be less critical for imaging applications, where the tumour‑to‑background ratio remains the primary determinant of performance, it becomes considerably more limiting when envisioning translation toward therapeutic applications. As the piperazine moiety can be potentially modified without affecting onalespib’s affinity for Hsp90, structural optimization at this position could be explored to improve the metabolic stability of the final radiotracer.

## Conclusions

The landscape of Hsp90-targeting radiotracers is rapidly evolving. This study demonstrated successful radiosynthesis of [^11^C]Onalespib and its feasibility for Hsp90 imaging in cancer. [^11^C]Onalespib exhibits Hsp90 specific uptake across three tumour types *in vitro* as well as in the brain tissues. Contrary to the previous reports, no brain uptake of the tracer was observed in mice, questioning the use of onalespib for glioblastoma applications. Its moderate tumour uptake in U-87 MG tumour xenograft was comparable to the previously developed Hsp90 tracers. Rapid metabolism of onalespib as observed by the presence of multiple radiometabolites in plasma is likely the reason for renal clearance besides hepatobiliary clearance. Further modifications of the tracer are planned to improve its metabolic stability and tumour accumulation, and recent reports on onalespib derivatisation highlight the feasibility of this promising approach.

## Supplementary Material

Supplementary Files

This is a list of supplementary files associated with this preprint. Click to download.

EJNMMISupplementalinformationLudovicLeSaux.pdf

## Data Availability

The datasets generated during and/or analysed during the current study are available from the corresponding author on reasonable request.

## List of abbreviations

%ID: Percentage injected dose
AD: Alzheimer’s disease
ATCC: American type culture collection
ATP: Adenosine triphosphate
BBB: Blood-brain barrier
BSA: Bovine serum albumin
CPM: Counts per minute
DIPEA: *N,N*-diisopropylethylamine
DLU/mm^2^: Digital light units per square mm
DMEM: Dulbecco’s Modified Eagle’s Medium
DMSO: Dimethylsulfoxide
ER: Endoplasmic reticulum
FBS: Fetal bovine serum
GRP94: 94 kDa glucose-regulated protein
HER2: Human epidermal growth factor receptor
HPLC: High-performance liquid chromatography
Hsp90: Heat shock protein 90
*i.p*.: Intraperitoneally
*i.v*.: Intravenously
LC-MS: Liquid chromatography-mass spectrometry
MIP: Maximum intensity projection
NMR: Nuclear magnetic resonance
PBS: Phosphate buffered saline
*p.i*.: Post-injection
PET: Positron emission tomography
PD: Parkinson’s disease
QC: Quality control
RCP: Radiochemical purity
RCY: Radiochemical yield
RP-HPLC: Reversed-Phase High-Performance Liquid Chromatography
SD: Standard deviation
SPECT: Single-photon emission computed tomography
SUV: Standardized uptake value
TAC: Time-activity curve
TRAP1: Tumour necrosis factor receptor-associated protein 1
Tris HCl: Tris(hydroxymethyl)aminomethane hydrochloride
